# Impact of harm minimization interventions on reducing blood-borne infection transmission and some injecting behaviors among people who inject drugs: an overview and evidence gap mapping

**DOI:** 10.1186/s13722-024-00439-9

**Published:** 2024-02-04

**Authors:** Fernanda S. Tonin, Filipa Alves da Costa, Fernando Fernandez-Llimos

**Affiliations:** 1 H&TRC - Health & Technology Research Center, ESTeSL - Escola Superior de Tecnologia da Saúde, Instituto Politécnico de Lisboa, Lisbon, Portugal; 2 Research Institute for Medicines (iMED.ULisboa), Faculty of Pharmacy, University of Lisbon, Av. Prof. Gama Pinto, Lisbon, Portugal; 3 Applied Molecular Biosciences Unit, (UCIBIO-i4HB) Laboratory of Pharmacology, Faculty of Pharmacy, University of Porto, Porto, Portugal

**Keywords:** Harm reduction, Injectable drug use, Blood-borne infections, Systematic reviews, Evidence gaps

## Abstract

**Background:**

This study aimed to synthetize the evidence on the effectiveness of harm minimization interventions on reducing blood-borne infection transmission and injecting behaviors among people who inject drugs (PWID) through a comprehensive overview of systematic reviews and evidence gap mapping.

**Methods:**

A systematic review was conducted with searches in PubMed and Scopus to identify systematic reviews assessing the impact of interventions aimed at reducing the harms associated with injectable drug use. The overall characteristics of the studies were extracted and their methodological quality was assessed using AMSTAR-2. An evidence gap map was constructed, highlighting the most frequently reported outcomes by intervention (CRD42023387713).

**Results:**

Thirty-three systematic reviews were included. Of these, 14 (42.2%) assessed the impact of needle/syringe exchange programs (NSEP) and 11 (33.3%) examined opioid agonist therapy (OAT). These interventions are likely to be associated with reductions of HIV/HCV incidence (10–40% risk reduction for NSEP; 50–60% for OAT) and sharing injecting paraphernalia (50% for NSEP, 25–85% for OAT), particularly when combined (moderate evidence). Behavioral/educational interventions were assessed in 12 reviews (36.4%) with most authors in favor/partially in favor of the use of these approaches (moderate evidence). Take-home naloxone programs and supervised-injection facilities were each assessed in two studies (6.1%), which reported inconclusive results (limited/inconsistent evidence). Most authors reported high levels of heterogeneity and risk of bias. Other interventions and outcomes were inadequately reported. Most systematic reviews presented low or critically low quality.

**Conclusion:**

The evidence is sufficient to support the effectiveness of OAT, NSEP and their combination in reducing blood-borne infection transmission and certain injecting behaviors among PWID. However, evidence of other harm minimizations interventions in different settings and for some outcomes remain insufficient.

## Introduction

Injecting drugs remains a substantial contributor to global morbidity and mortality. While the patterns of drugs injected have changed, injecting behaviors have, in general, been on the decline in several regions for the past decade. Globally, out of the more than 12 million people who inject drugs (PWID), approximately 6 million are living with acquired blood-borne infectious diseases, particularly caused by the human immunodeficiency virus—HIV (accounting for 12.5% of new infections) and hepatitis B and C virus (HBV, HCV) (20–40% of cases) [1–3]. Unsafe drug practices among PWID are also associated with higher risk of overdose and drug-related fatalities [1, 2]. It is estimated that 20.5% (95% CI, 15.0–26.1%) and 41.5% (95% CI, 34.6–48.4%) of PWID have experienced at least one non-fatal overdose in the previous 12 months and in their lifetime, respectively [4]. Pooled crude mortality rates among PWID are 2.35 deaths per 100 person-years (95% CI 2.12–2.58) [5]. Furthermore, drug use places additional burdens on PWID and the society at large, including healthcare costs, efforts to combat crime, and lost productivity, [6].

Over the past 35 years, many countries have developed policy and public health initiatives aimed at addressing the health, societal, and economic adverse consequences of drug use [7, 8]. In addition to outreach programs and rehabilitation clinics, strategies involving a range of providers have been implemented globally to reduce or minimize the harms associated with drug use for individuals who are not prepared to quit. [7, 9]. Some of the most common harm minimization interventions include: (i) providing naloxone (i.e., naloxone dispensing without an external prescription through take-home naloxone programs – THN); (ii) opioid agonist or substitution therapies – OAT/OST, including medications for opioid use disorders (MOUD) such as methadone; (iii) supply-reduction interventions for opioids (e.g., prescription monitoring programs, tamper-resistant formulations, and prescribing limits); (iv) non-prescription sales or provision of sterile syringes through needle and syringe exchange programs (NSEP), including within supervised drug consumption facilities or supervised injection facilities (SCF or SIF); and (v) integration of testing and treatment for blood-borne diseases through screening and point-of-care testing (i.e., diagnostic testing conducted outside a laboratory environment, generally at or near to the patient’s location) [10].

The literature is abundant with studies, including a dozen systematic reviews and meta-analyses that have assessed the impact of these interventions in improving drug-related harms. However, substantial variations in methods, outcome measures, and transparency of their reporting exist, which can lead to results suggesting interventions that may not be effective. Moreover, when interventions are implemented, they may not be adequately translated into practice or tailored to suit the relevant populations. While there are a limited number of literature overviews (i.e., systematic reviews of systematic reviews) available, they tend to focus on specific interventions (e.g., NSEP) and outcomes (e.g., HIV-related harms). These overviews are often outdated (last publications from 2022 including primary studies published until 2019–2020) and tend to concentrate on the broader category of people who use drugs. Furthermore, they may not thoroughly evaluate the roles of PWID and stakeholders on harm minimization initiatives (e.g., barriers to implementation and upscale) [11–16].

Considering the persistent global drug use crisis, coupled with strained healthcare resources and growing associated burdens, a pressing need for the implementation of higher-quality, scaled up evidence-based approaches in this field exists. These approaches are essential to facilitate informed decision-making and the development of strategies for future policy planning. This study aimed to answer the question ‘What is the extent and current state of evidence regarding the effectiveness of harm minimization interventions in reducing blood-borne infection transmission and injecting behaviors among PWID?’ This was accomplished through a comprehensive and up-to-date overview of systematic reviews and evidence gap mapping.

## Methods

A systematic review of systematic reviews (overview or umbrella review) was conducted following the Joanna Briggs Institute and the Cochrane recommendations and reported following the Preferred Reporting Items for Systematic Reviews and Meta-Analyses (PRISMA) statement [17–19]. (Protocol PROSPERO—CRD42023387713).

### Search and eligibility criteria

A systematic search was conducted in PubMed and Scopus without timeframe or language limits (updated December 2022). In addition, a manual search was performed in the reference lists of included studies, and conventional search engines (i.e., Google and Google Scholar). Search strategies are available in the Supplementary Material.

Studies retrieved were organized into Endnote X7 and duplicate records removed. Two reviewers conducted independent screening (title/abstract reading) and full-text evaluation using Microsoft Excel 2013. Data extraction and methodological quality assessment for the included studies were performed by a single reviewer and verified by another. Discrepancies were addressed through discussion involving a third reviewer.

This review included articles: (i) published peer-reviewed systematic reviews (with or without meta-analysis) that included primary studies of any design (interventional, observational); (ii) aimed at assessing the effects of any intervention, method, or approach (i.e., program, service, or study) provided by any professional with the aim of reducing or minimizing harm associated with injectable drug use in any setting; and (iii) provided results related to the reduction or alterations of risk behaviors’ outcomes, such as illicit opioid use, overdose, drug-related fatalities, injecting behavior, sharing of needle/syringe or equipment, as well as HIV and HCV incidence or prevalence rates. Study protocols, overviews, articles that were restricted to people who use drugs without differentiation of results for PWID, and studies written in non-Roman characters were excluded.

### Data extraction and quality assessment

To collect data, we used a standardized data collection form. This form was used to extract: general characteristics of the reviews (authors, publication year, sample size [number and design of included studies]); type of intervention and comparator (when available); setting; main reported outcomes and results.

The methodological quality of the studies was evaluated using the A MeaSurement Tool to Assess systematic Reviews (AMSTAR-2), designated to assess the quality of the systematic reviews of both randomized and non-randomized studies of health interventions [20]. AMSTAR-2 comprises 16 domains, and for each domain reviewers provide answers among ‘yes’, ‘partial yes’, ‘no’, ‘not applicable’. The quality rating is determined by identifying the weaknesses in the critical domains, and the final score enables grading and ranking methodological quality, ranging from ‘critically low’ to ‘high’.

### Data synthesis and evidence gap mapping

The individual results of the studies and effect-size measures were summarized considering the information provided by the authors. The outcomes were measured using different effect size metrics, including: odds ratio (OR), adjusted odds ratio (aOR), standardized mean difference (SMD), risk ratio (RR), weighted mean (WM). Confidence interval (CI) of effect size measures was collected, along with the information about the level of statistical significance and heterogeneity between studies (I^2^ index) when reported.

The findings were ultimately synthesized into an evidence gap map, considering the most frequently reported outcomes and the methodological quality of the systematic reviews for each intervention. This approach provides a visual summary of the breadth and availability of information within a specific area and the gaps in the current evidence, which may ground further research, and policy development [21].

## Results

### Studies’ overall characteristics and methodological quality

**Fig. 1:**
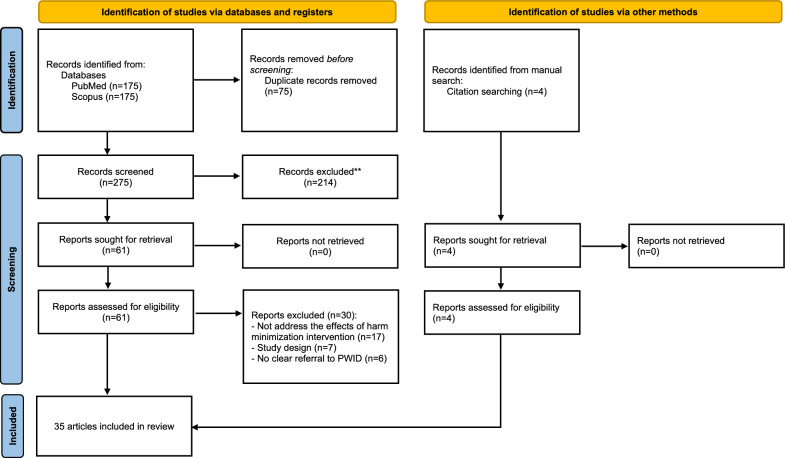
Flowchart of the systematic review

A total of 275 records were retrieved from the databases after duplicates removal. Following screening, 61 articles were considered for full-text analysis, and 31 studies met the eligibility criteria for data extraction. Four studies were added through manual searches, totaling 35 included studies (see Fig. 1) [22–56]. Excluded studies are available in the Supplementary Material.

The 35 articles report 33 systematic reviews published between 1998 and 2021, of which16 (48.5%) are qualitative systematic reviews, and 17 (51.5%) include statistical analyses (i.e., meta-analysis or meta-synthesis). These systematic reviews encompassed various primary study designs, most of which assessed as having low-moderate methodological quality (i.e., moderate or high risk of bias). The most frequently evaluated outcomes were the incidence and prevalence of HIV and HCV, overall risk behavior (including sexual risk behavior, injecting behavior or drug use), injecting behavior (including reusing of syringes, injecting outdoors, and rushing injections), injection drug use, sharing of needles/syringes, and illicit opioid use. Conversely, assessments of drug treatment entry, overdose rates, and drug-related fatalities were limited (Table 1).

**Table 1:** Characteristics of systematic reviews on the impact of harm minimization interventions (n = 33)

Author	Study design	Included primary studies	Setting	Interventions	Main outcomes* (pooled effect-size [95% CI])	Primary studies’ quality	Conclusions	Favors the intervention? #
*NSEP approach*								
Abdul-Quader [22]	SLR	15 interventional studies	Any health setting	NSEP	• HIV prevalence: N = 12/15 studies (80%) show reductions of − 0.6% to − 43% • HCV prevalence: N = 5/7 studies (71%) show reductions of − 13% to − 30%	Moderate	Results support NSEP as a structural-level intervention to reduce HIV and HCV infections	Yes
Aspinall [23]	SLRMA	12 observational studies (n = 12,000)	Pharmacies Outreach services	NSEP	• HIV transmission, *all studies*: RR 0.66 [0.43–1.01], I2 = 76% • HIV transmission, *high quality studies*: RR 0.42 [0.22–0.81], I2 = 79%	Moderate	NSEP is effective in reducing HIV transmission, although other harm reduction interventions can contribute to this	Yes
Davis [24]	SLRMA	6 observational studies (n = 2437)	Any health setting	NSEP	• Association of HCV seroconversion and NSEP participation: OR 0.51 [0.05–5.15], I2 = 88%	Low	Evidence is mixed and inconclusive; no consistent association on NSEP impact on HCV was found	Inconclusive
Des Jarlais [25]	SLR	11 interventional and observational studies	Low- and middle-income settings	NSEP	• HIV prevalence: N = 5/8 studies (63%) show reductions of − 3% to − 15% • HCV prevalence: N = 3/4 studies (75%) show reductions of − 4.2% to − 10.2%	–	While not fully consistent and homogeneous, overall evidence support the effectiveness of NSEP in reducing HIV/HCV in these countries	Yes
Gibson [26]	SLR	42 interventional and observational studies	Any health setting	NSEP	• Sharing needles/syringes: N = 28/42 studies (67%) show reductions	–	Studies show a slightly benefit of NSEP; but methodologic rigor needs to be improved (true impact of the interventions: unknown)	Inconclusive
Gillies [27]	SLR	13 observational studies	Outreach services, SCF/SIF	NSEP	• Sharing needles/syringes: aOR ranging from 0.3 to 0.9	Low	Studies suggest a reduced likelihood of sharing paraphernalia. However, estimates are uncertain given studies’ low quality	Inconclusive
Jones [28]	SLR	16 RCT or observational studies	Any health setting	NSEP	• Injecting behavior: not statistically significant • BBV incidence/prevalence: not statistically significant • Drug treatment entry: not statistically significant	–	Most studies (n = 11) showed no evidence of impact of NSEP or syringe dispensation policies on injecting behaviors. Studies are heterogeneous	No
Ksobiech [29]	SLRMA	31 observational studies(n = 52,678)	Any health setting	NSEP	• Injection drug use: Weigh. correlat − 0.189 (SE 0.05) • Injection frequency: Weigh. correlat: − 0.024 (SE 0.04) • Sharing needles/syringes: Weigh. correlat: − 0.059 (SE 0.02) • Risky behavior: Weigh. correlat: + 0.016 (SE 0.07)	–	NSEP attendance was inversely related to the reduction of most harmful outcomes, however, given the high heterogeneity among studies, data should be carefully interpreted	Partially yes
Sawangjit [30]	SLRMA	14 observational studies (n = 7035)	Pharmacies	NSEP	• Sharing needles/syringes, *all studies*: OR 0.50 [0.34–0.73], I2 = 60% • Sharing needles/syringes, *high quality studies*: OR 0.52 [0.32–0.84], I2 = 41% • HCV prevalence: OR 0.26 [0.18–0.38], I2 = 0% • HIV prevalence: OR 0.56 [0.18–1.77], I2 = 92.7%	Low-moderate	Pharmacy-based NSEP programs appear to be effective for reducing risk behaviors, but their effect on HIV/HCV prevalence and economic outcomes are still unclear	Partially yes
*OAT approach*								
Gowing [31]	SLR	38 interventional studies (n = 12,400)	Any health setting	OAT	• Injection drug use: Reduced by 20–60% • Illicit opioid use: Reduced by 32–69% • Sharing needles/syringes: Reduced by 25–86%	Low	OAT may reduce drug-related behaviors with a high risk of HIV transmission. The lack of data from RCT limits the strength of the evidence	Yes
Hedrich [32]	SLR	21 RCT or observational studies	Prisons	OAT	• HCV or HIV incidence: not statistically significant • Drug treatment entry: significant positive effect of OAT • Sharing needles/syringes: significant positive effect of OAT • Injecting behavior: significant positive effect of OAT • Illicit opioid use: significant positive effect of OAT	Low-moderate	The evidence is overall consistent and supports the use of OAT/OST to risky behaviors. Yet, for some outcomes, evidence is inconsistent (crime, re-incarceration) or weak (HCV incidence, mortality)	Partially yes
Karki [33]	SLR	12 RCT or observational studies (n = 16,195)	Any health setting	OAT	• Risky behavior reduction: significant positive effect of MOUD/OAT	–	MOUD is associated with significant decrease in injecting drug use and sharing of injecting equipment. Evidence for other outcomes limited	Partially yes
Larney [34]	SLR	5 interventional studies	Prisons	OAT	• Injection drug use: Reduced by 55–75% • Sharing needles/syringes: Reduced by 47–73% • Illicit opioid use: Reduced by 62–91%	Low-moderate	There may be a role for OAT in preventing HIV in prisons, but rigorous research addressing this question is required	Yes
MacArthur [35]	SLRMA	12 observational studies	Any health setting	OAT	• HIV transmission: RR 0.60 [0.42–0.85], I2 = 23% • HIV incidence: RR 0.46 [0.32–0.67], I2 = 60%	Low-moderate	OAT by means of MOUD is associated with reduction in the risk of HIV infection among PWID, although some heterogeneity/bias among studies exist	Yes
Moore [36]	SLRMA	24 interventional studies (n = 807)	Prisons	OAT	• Injection drug use: OR 0.26 [0.12–0.56], I2 = 62% • Illicit opioid use: OR 0.22 [0.15–0.32], I2 = 0% • Recidivism: OR 0.93 [0.51–1.68], I2 = 46%	Moderate	Data support the use of MOUD/OAT in prisons for reducing some outcomes; yet, additional work is needed to understand the its impact on other health risk behaviors	Partially yes
Behavioral or educational interventions								
Copenhaver [37]	SLRMA	37 RCT (n = 10,190)	Any health setting	Behavioral interventions	• Injection drug use: WM 0.08 [0.03–0.13], p < 0.001 • Drug treatment entry: WM 0.11 [0.02–0.21], p = 0.013 • Sharing needles/syringes: WM 0.03 [− 0.04, 0.10],p = 0.062 • Frequency of trading sex for drugs: WM 0.33 [0.10–0.57], p = 0.052	–	Behavioral interventions were effective in reducing some HIV-risk behaviors; yet no benefits were observed for reducing needle or syringe borrowing	Partially yes
Deuba [38]	SLRMA	43 RCT (n = 15,642)	Any health setting in low-income countries	Behavioral interventions (*peer-based*)	• HIV prevalence, *PWID studies*: 11.9% [8.3–16.7] • Unsafe injections, *PWID studies*: OR 0.942 [0.726–1.222], I2 = 0%	Moderate	None of the included interventions were found to be effective for reducing unsafe injection practices among PWID in low-income countries	No
Gilchrist [39]	SLRMA	24 RCT (n = 12,840)	Any health setting	Psychosocial interventions	• Any injecting behavior: SMD − 0.29 [− 0.42, − 0.15], I2 = 61% • Sharing needles/syringes: SMD − 0.43 [− 0.69, − 0.18], I2 = 68% • Injecting drug use: SMD − 0.17 [− 0.35, 0.00], I2 = 61% • Sexual risk behavior: SMD − 0.19 [− 0.30, 0.01], I2 = 58% • HIV testing/counseling: SMD − 0.24 [− 0.44, − 0.03], I2 = 0%	Low-moderate	Psychosocial interventions appear to reduce some risky behavior outcomes, but moderate heterogeneity was reported. Such interventions can be used to prevent BBV	Partially yes
Meader [40]	SLRMA	35 interventional studies (n = 11,867)	Any health setting	Psychosocial interventions Educational interventions	• Injection risk behaviour SMD − 0.04 [− 0.31, 0.23], I2 = 69% • Sexual risk behavior: SMA − 0.12 [− 0.33, 0.08], I2 = 49%	Low-moderate	Both multi-session psychosocial interventions and standard education can reduce injection and sexual risk behaviour; minimal differences between interventions were found	Yes
Prendergast [41]	SLRMA	18 controlled studies	Any health setting	Behavioral interventions	• HIV risk-reduction, *overall:* WM 0.31 [0.20–0.42], p < 0.01 • Injection drug use: WM 0.04 [− 0.14, 0.22], p > 0.05 • Sexual risk behavior: WM 0.26 [0.15–0.38], p < 0.01	–	The overall effect sizes suggests that HIV interventions within drug treatment have a reliable effect. Yet, heterogeneity among studies is high	Partially yes
Sacks-Davis [42]	SLR	6 RCT (n = 5472)	Any health setting	Behavioral interventions	• HCV incidence: not statistically significant • Sexual risk behavior: not statistically significant • Injecting behavior: significant positive effect of intervention	Low-moderate	Behavioral approach can have some effects on HCV transmission; yet considerable variations in study design, outcomes, magnitude/direction of effect exist	Inconclusive
Semaan [43]	SLRMA	33 interventional studies	Any health setting in the United States	Behavioral interventions	• Sexual risk behavior: OR 0.86 [0.76–0.98], p < 0.05, I2 = 47%	–	Behavioral-based interventions may lead to reduction on sexual risk behavior among drug users, but data is heterogeneous	Yes
*SCF/SIF studies*								
Kennedy [44]	SLR	47 observational studies	SCF/SIF	SCF/SIF	• Overdose mortality/morbidity: N = 6/8 studies (75%) show reductions • Risky behavior: N = 4/9 studies (44%) show reductions	–	These facilities may mitigate overdose-related harms and unsafe drug use. Yet, no meta-analyses were performed; outcomes are not standardized	Inconclusive
Levengood [45]	SLR	22 observational studies	SCF/SIF	SCF/SIF	• Overdose mortality/morbidity: N = 4/5 studies (80%) show reductions • Injecting behavior: N = 6/7 studies (86%) show reductions • Drug treatment entry: N = 6/7 studies (86%) show increases • Crime and public nuisance: N = 5/7 studies (70%) show stability	Low-moderate	These facilities may reduce overdose-related risks and improve access to care, while not increasing crime or publicnuisance to the surrounding community. Inconsistent outcomes across studies prevent further conclusions	Inconclusive
*THN approach*								
McAuley [46]	SLRMA	9 observational studies	SCF/SIF Drug treatment centers Prisons	THN	• Proportion of naloxone use (every 3 months / 100 trained users): WM: 0.092 [0.052–0.131]	–	Around 9% of naloxone kits distributed are likely to be used for peer administration within three months’ supply. The evidence for THN is limited	Inconclusive
McDonald [47]	SLR	22 observational studies (n = 2912)	Any health setting	THN	• Overdose reversals: 96.3% [95.5–97.1] (n = 2249/2336 THN administrations) • Deaths: 0.9% [0.5–1.2] (n = 24/2336 THN administrations)	Low-moderate	THN programmes can reduce overdose mortality with a low rate of adverse events. However, there is a large variability in the size and quality studies	Inconclusive
*Other combined interventions and interventions’ comparisons*								
Bouzanis [48]	SLR	97 interventional and observational studies	Any health setting in Canada	NSEP SCF/SIF OAT PoC Behavioral interventions	Evidence indicates advantages of multifaceted care programmes for PWID, which include harm reduction, medical/pharmaceutical treatments, social support and education	–	The included studies call for exploratory work in facilitators and barriers to treatment and care, more robust study designs, and attention to contextual factors and more complex interventions	Inconclusive
Cross [49]	SLRMA	18 interventional studies (n = 7926)	Any health setting	Educational interventions NSEP	• Risky behavior, *educational studies*: WM: 0.749 [0.708–0.790] • Risky behavior, *NSEP studies:* WM: 0.279 [0.207–0.352]	–	Both interventions had a positive impact on reducing HIV risk behaviors, however, results are dependent upon research design, outcomes, follow-up	Yes
Hagan [50]	SLRMA	26 interventional and observational studies	Any health setting	NSEP OAT Behavioral interventions	• HCV incidence, *behavioral studies:* RR: 1.18 [0.77–1.81], I2 = 0% • HCV incidence, *OAT studies:* RR: 0.60 [0.35–1.03], I2 = 45% • HCV incidence, *NSEP studies:* RR: 1.62 [1.04–2.52], I2 = 81% • HCV incidence, *multi-component:* RR: 0.25 [0.07–0.83], I2 = 55%	Moderate	Multi-component interventions using strategies that combined substance-use treatment and support for safe injection were most effective at reducing HCV seroconversion	Yes
McNeil [51]	SLR	21 qualitative studies (n = 800)	Urban or semi-urban settings	NSEP SCF/SIF Behavioral interventions (*peer-based*)	Interventions are potentially associated with: (1) providing refuge from streets (2) enabling safer injecting environments (3) mediating access to resources and health care services (4) constrained by drug prohibition and law enforcement activities	–	Safer environment interventions may mitigate drug-related harms. Further qualitative and quantitative evidence syntheses in this field are needed	Inconclusive
Platt 2017, [52–54]	SLRMA	28 observational studies (n = 6279)	Any health setting	NSEP OAT	• HCV incidence, *all studies:* RR 0.26 [0.07–0.59], I2 = 80% • HCV incidence, *OAT studies:* RR 0.50 [0.40–0.63], I2 = 0% • HCV incidence, *NSEP studies:* RR 0.79 [0.39–1.61], I2 = 77%	Low-moderate	Although the evidence is still of low quality and should be strengthened, it seems that the combination of OAT and NSEP significantly reduce the risk of HCV acquisition	Partially yes
Turner [55]	SLRMA	6 individual-level data studies (n = 2986)	Any health setting	NSEP OAT	• HCV incidence, *all studies:* aOR 0.52 [0.32–0.83] • HCV incidence, *OAT studies:* aOR 0.41 [0.21–0.82], I2 = 48% • HCV incidence, *NSEP studies:* aOR 0.48 [0.25–0.93], I2 = 0% • Injection frequency, *all studies*: − 20.8 [− 27.3, − 14.4] injections/month	Moderate	OAT and high coverage NSEP substantially reduced the risk of HCV transmission among injecting drug users (full harm reduction of needle sharing by 48% and mean injecting frequency by 20 injections per month)	Yes
Wright [56]	SLR	18 interventional and observational studies	Any health setting	NSEP OAT Behavioral interventions	• HCV incidence, *NSEP studies:* significant positive effect NSEP • HCV incidence, *OAT studies:* significant positive effect of OAT • Limited evidence evaluating behavioural interventions	–	NSEP or OAT primary interventions are marginally effective in reducing HCV prevalence. Further combined interventions should be assessed	Yes

^*^Data related only to harm minimization or harm reduction interventions. Results are displayed as reported by authors (whenever possible as pooled effect size with 95% CI)

^#^Overall interpretation of the findings that favors the use of the interventions to reduce harms (yes, no, partially [i.e., benefits for some of the outcomes], inconclusive [i.e., no conclusions can be drawn on interventions’ benefits or otherwise due to inconsistent evidence]

BBV: blood borne viral infections; CI: confidence interval; HCV: hepatitis C; HIV: human immunodeficiency virus; I2: between-studies heterogeneity index; MOUD: medication assisted treatment [methadone, buprenorphine, or naltrexone]; NSEP: needle and syringe exchange program; PoC: point-of-care testing; PWID: people who inject drugs; RR: risk ratio; OR: odds ratio; aOR: adjusted odds ratio; OMT: opioid maintenance treatment; OAT: opioid agonist maintenance treatment; OST: opioid substitution therapy; RCT: randomized controlled trial; SCF: supervised drug consumption facilities; SIF: supervised injection facility; SMD: standard mean difference; SLR: systematic literature review; SLRMA: systematic literature review and meta-analysis; THN: take-home naloxone program; WM: weighted mean effect size; Weigh. correlat: weighted correlation

The methodological quality assessment of the included systematic reviews using AMSTAR 2 is summarized in Table 2. Most studies (n = 17; 51.5%) presented low quality. Shortcomings included instances where authors did not: provide the list of excluded studies and justified these exclusions (item 7); employ a technique for assessing the risk of bias in primary studies (item 9); report the sources of funding (item 10); account for risk of bias in individual studies when interpreting or discussing the results (item 13); offer explanation for, or discussion of, observed heterogeneity in the results (item 14). In contrast, 6 studies (18.2%) were rated as having high methodological quality. These studies reported various interventions, including behavioral or psychosocial interventions [39, 42], NSEP [23, 24], OAT [35], and both NSEP and OAT [53, 54]).

**Table 2:** Methodological quality assessment (AMSTAR-2) of the included systematic reviews (n = 33)

Author	Item 1	Item 2	Item3	Item 4	Item 5	Item 6	Item 7	Item 8	Item 9	Item 10	Item 11	Item 12	Item 13	Item 14	Item 15	Item 16	Final rate (quality of the review)
Abdul-Quader [22]	Y	PY	Y	PY	Y	Y	PY	Y	Y	Y	N/A	N/A	Y	Y	N/A	Y	Moderate
Aspinall [23]	Y	PY	Y	Y	Y	Y	PY	Y	Y	Y	Y	Y	Y	Y	Y	Y	High
Bouzanis [48]	Y	PY	Y	PY	Y	Y	N	PY	N	N	N/A	N/A	N	N	N/A	Y	Critically low
Copenhaver [37]	Y	PY	Y	PY	Y	Y	N	PY	N	N	Y	N	N	N	Y	Y	Critically low
Cross [49]	Y	PY	Y	PY	N	N	PY	PY	N	N	Y	N	N	N	Y	Y	Low
Davis [24]	Y	Y	Y	Y	Y	Y	PY	Y	Y	Y	Y	Y	Y	Y	Y	Y	High
Des Jarlais [25]	Y	PY	Y	PY	Y	Y	N	PY	N	N	N/A	N/A	N	N	N/A	Y	Critically low
Deuba [38]	Y	PY	Y	PY	Y	Y	PY	Y	Y	Y	Y	Y	Y	Y	N	Y	Moderate
Gibson [26]	Y	PY	Y	PY	Y	Y	N	PY	N	N	N/A	N/A	N	N	N/A	Y	Critically low
Gilchrist [39]	Y	Y	Y	Y	Y	Y	PY	Y	Y	Y	Y	Y	Y	Y	Y	Y	High
Gillies [27]	Y	PY	Y	PY	Y	Y	PY	Y	Y	Y	N/A	N/A	Y	Y	N/A	Y	Moderate
Gowing [31]	Y	PY	Y	PY	Y	Y	PY	Y	Y	Y	N/A	N/A	Y	Y	N/A	Y	Moderate
Hagan [50]	Y	PY	Y	PY	Y	Y	PY	PY	PY	Y	Y	Y	Y	Y	Y	Y	Moderate
Hedrich [32]	Y	PY	Y	PY	Y	Y	N	PY	PY	N	N/A	N/A	N	N	N/A	Y	Low
Jones [28]	Y	PY	Y	PY	Y	Y	N	PY	Y	N	N/A	N/A	N	N	N/A	Y	Low
Karki [33]	Y	PY	Y	PY	Y	Y	N	PY	N	N	N/A	N/A	N	N	N/A	Y	Critically low
Kennedy [44]	Y	PY	Y	PY	Y	Y	N	PY	N	N	N/A	N/A	N	N	N/A	Y	Critically low
Ksobiech [29]	N	N	N	N	N	N	N	Y	N	N	Y	N	N	Y	N	Y	Critically low
Larney [34]	Y	PY	Y	N	Y	Y	N	PY	Y	N	N/A	N/A	N	Y	N/A	Y	Low
Levengood [45]	Y	PY	Y	PY	Y	Y	PY	Y	PY	Y	N/A	N/A	N	Y	N/A	Y	Moderate
MacArthur [35]	Y	PY	Y	PY	Y	Y	N	Y	Y	Y	Y	Y	N	Y	Y	Y	High
McAuley [46]	Y	PY	N	N	N	N	N	PY	N	N	Y	N	N	N	N	Y	Critically low
McDonald [47]	Y	Y	Y	Y	Y	Y	N	PY	PY	Y	N/A	N/A	Y	N	N/A	Y	Moderate
McNeil [51]	Y	PY	Y	PY	N	N	N	PY	N	N	N/A	N/A	N	N	N/A	Y	Critically low
Meader [40]	Y	PY	Y	Y	Y	Y	Y	Y	Y	Y	Y	Y	N	N	N	Y	Moderate
Moore [36]	Y	PY	N	PY	Y	Y	N	PY	Y	Y	Y	Y	N	N	N	Y	Low
Platt [52–54]	Y	PY	Y	Y	Y	Y	Y	Y	Y	Y	Y	Y	Y	Y	N	Y	High
Prendergast [41]	N	N	N	N	N	N	N	Y	N	N	Y	N	N	Y	N	Y	Critically low
Sacks-Davis [42]	Y	PY	Y	Y	Y	Y	N	Y	Y	Y	N/A	N/A	Y	Y	N/A	Y	High
Sawangjit [30]	Y	Y	Y	PY	Y	Y	PY	Y	Y	Y	Y	Y	Y	N	N	Y	Moderate
Semaan [43]	N	N	N	N	N	N	N	Y	N	N	Y	N	N	N	N	Y	Critically low
Turner [55]	Y	PY	Y	N	Y	Y	N	PY	Y	Y	Y	Y	Y	N	N	Y	Moderate
Wright [56]	Y	N	Y	PY	Y	Y	N	PY	N	N	N/A	N/A	N	N	N/A	Y	Critically low

N: no; PY: partial yes; Y: yes; N/A: not applicable (no meta-analysis performed). Itens 1–16 are fully described in *AMSTAR 2: a critical appraisal tool for systematic reviews that include randomised or non-randomised studies of healthcare interventions (Shea BJ *et al*. BMJ. 2017 Sep 21;358:j4008;* https://amstar.ca/index.php

### NSEP and OAT

Approximately one-third of studies (n = 9; 27.3%) exclusively evaluated the impact of NSEP on PWID. Five of these had moderate or high methodological quality, while four had critically low or low methodological quality. OAT on its own was assessed in six reviews (18.2%), two with moderate or high methodological quality and four with critically low or low quality. Five studies (12.1%) compared the effects of NSEP, OAT, and their combination in reducing drug-injecting related harms. Among these, three had moderate or high methodological quality, and two had critically low quality. The most frequently reported outcomes were related to the transmission of HIV/HCV.

Considering authors’ conclusions, most studies were in favor or partially in favor of these interventions (i.e., benefits for some outcomes) in various settings. Specifically, 71.4% of the studies on NSEP and 100% on OAT indicated positive results, including benefits in reducing HIV transmission and prevalence, HCV prevalence, and risk behaviors such as sharing needles/syringes (Table 1). Only one review (Jones et al. 2010) [28] (low methodological quality) concluded that there were no significant benefits from NSEP (outcomes of injecting behavior, incidence of blood borne viral infections and drug treatment entry). Three other studies (21.4%), two with high or moderate methodological quality, and one with critically low quality, were inconclusive. In these reviews, there was no consistent association between NSEP and its impact, primarily due to high between-studies heterogeneity, low methodological quality, and inconsistent data (e.g., non-standardized outcomes to enable comparisons)[24, 26, 27].

According to the meta-analyses performed by Hagan et al. [50], Platt et al. [52–54], and Turner et al. [55] (respectively moderate, high and moderate methodological quality), the use of OAT was associated with significant reductions in HCV incidence. RR ranged from 0.60 [95% CI 0.35–1.03] (I^2^ = 45%) to RR 0.50 [95% CI 0.40–0.63] (I^2^ = 0%). Additionally, an aOR of 0.41 [0.21–0.82], was reported. In contrast, NSEP yielded less significant and yet more heterogeneous results for this outcome (results ranged from RR 1.62 [95% CI, 1.04–2.52] (I^2^ = 81%) to RR 0.79 [95% CI 0.39–1.61] (I^2^ = 77%) and an aOR 0.48 [95% CI 0.25–0.93]). These meta-analyses indicated that multi-component interventions (e.g., NSEP and OAT) can contribute to a substantial reduction in the risk of acquiring HCV (around 75–80%). The authors suggested that this reduction is likely attributable to the OAT component.

Aspinall et al. [23] and MacArthur et al. [35] (both high methodological quality) reported significant reductions of HIV transmission with NSEP and OAT in health settings (RR 0.42 [95% 0.22–0.81] (I^2^ = 79%), and RR 0.60 [95% 0.42–0.85] (I^2^ = 23%), respectively). However, Hedrich et al. [32] (low methodological quality) found no significant effects of OAT on HCV/HIV incidence in prison settings.

Sawangjit et al. [30] and Cross et al. [49] (respectively moderate and low methodological quality) reported favorable results of NSEP on reducing sharing needles/syringes (OR 0.50 [95% CI 0.34–0.73]; I^2^ = 60%) and overall risk behaviors (weighted mean 0.279 [95% CI 0.207–0.352]). Reductions in sharing paraphernalia, ranging from 25 to 86%, were observed by Gowing et al. [31] and Larney et al. [34] (respectively moderate and low methodological quality) following the implementation of OAT. According to the meta-analysis by Moore et al. 2019 (low methodological quality), OAT was also significantly associated with a reduction in illicit opioid use (including in prisons) [36] (OR 0.22 [95% CI 0.15–0.32], I^2^ = 0%). These findings were consistent with Gowing et al. [31] and Larney et al. [34] with reduction rates ranging from 32 to 91%.

### Behavioral and psychosocial interventions

Behavioral, psychological, or educational/engagement interventions were focused in seven studies (21.2%) (four with moderate or high and three with critically low methodological quality). Five additional studies compared the effectiveness of these interventions with other approaches (e.g., OAT) (one with moderate and four with critically low or low methodological quality). Most authors (75.0%) were in favor of the use of behavioral interventions for reducing at least one injecting behavior in PWID.

Deuba et al. [38] (moderate methodological quality) concluded that behavioral, psychological, and educational/engagement strategies were not effective for reducing unsafe injection practices and HIV prevalence among PWID, especially in low-income settings. In contrast, a systematic review by Sacks-Davis et al. 2012 (high methodological quality) [42] suggested that behavioral approaches may have some effects on reducing HCV transmission. However, the review noted significant variations among observational studies in terms of design, outcomes, magnitude/direction, and statistical significance, which resulted in inconclusive data. Gilchrist et al. [39] and Semaan et al. [43] (respectively high and critically low methodological quality) found evidence that psychological and behavioral strategies were associated with reductions in sexual risk behaviors (SMD − 0.19 [95% CI − 0.30, 0.01], I^2^ = 58%, and OR 0.86 [95% CI 0.76–0.98]; I^2^ = 47%, respectively), and sharing paraphernalia (SMD − 0.43 [95% CI, − 0.69 to − 0.18], I^2^ = 68%). However, a high level of between-studies heterogeneity was reported. Other authors did not find significant results favoring these interventions.

### SCF/SIF

Naloxone access programs (i.e., THN) and supervised facilities (SCF or SIF) were evaluated in only two studies (6.1%) each, all of which reported inconclusive results on the benefits for PWID. This inconclusiveness is caused by limited data, such as the availability of few observational studies [ranging from five to nine] for each outcome of interest, inconsistent comparisons (THN or SIF vs. various controls), and variations in outcomes across primary studies. Kennedy et al. 2017 and Levengood et al. 2021 (respectively critically low and moderate methodological quality) [44, 45] suggested that supervised facilities might mitigate some overdose-related harms, with around 75–80% of studies reporting a reduction in related morbidity and mortality rates, and a reduction in unsafe drug use (approximately 85% reported a reduction of injecting behaviors). However, other outcomes including sexual risk behaviors and crime or public nuisance in the surrounding community require further medium and long-term studies. Similarly, evidence by McAuley et al. [46] and McDonald et al. [47] (respectively critically low and moderate methodological quality) from observational studies demonstrated that THN programs may reduce overdose mortality with a low rate of adverse events, but other outcomes in this setting are poorly reported. This evidence is further limited by the evaluation design and number of successful reversals. Only one systematic review without meta-analysis by Bouzanis et al. [48] (critically low methodological quality) mentioned the use of point-of-care HIV/HCV testing and treatment interventions, such as integrated multidisciplinary HIV and HCV care, supportive housing models, and addiction treatments for this population. Results should be carefully approached given the limitations in primary studies, including lack of randomization, self-reported measurements, and challenges in data generalizability (i.e., experiences often specific to PWID).

Table 3 summarizes the findings of this overview by means of an evidence gap map. The most reported outcomes were categorized as: HIV incidence/transmission, HIV prevalence, HCV incidence/transmission, HCV prevalence, overall risk behavior, illicit opioid use, injecting behavior, injection drug use, sharing needles/syringes, drug treatment entry, overdose, and deaths.

**Table 3:**
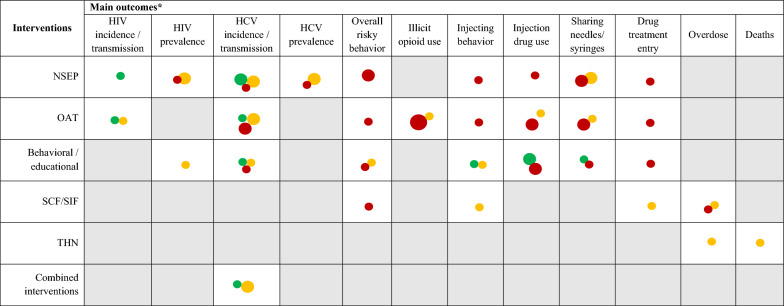
Summary of findings: evidence gap map

The size of the circles is proportional to the number of systematic reviews (larger = 3 or more; medium = 2; small = 1). Green circle: high methodological quality systematic review; yellow circle: moderate methodological quality systematic review; red circle: critically low or low methodological quality systematic review

HCV: hepatitis C; HIV: human immunodeficiency virus; NSEP: needle and syringe exchange program; OAT: opioid agonist maintenance treatment; SCF: supervised drug consumption facilities; SIF: supervised injection facility; THN: take-home naloxone program

^*^Outcomes:

HIV incidence/transmission: refers to rates or reported data on infection incidence and transmission

HIV prevalence: refers to disease prevalence in the population

HCV incidence / transmission: refers to rates or reported data on infection incidence and transmission

HCV prevalence: refers to disease prevalence in the population

Overall risky behavior: refers to any kind of harmful behavior, including sexual risk behavior and related outcomes, injecting behavior or drug use

Illicit opioid use: refers to the injection of opioid

Injecting behavior: refers to all of those related to injection practices as reusing or sharing syringes/needles, injecting outdoors [public drug use], rushing injections

Injection drug use: refers specifically to the use of drugs through injections (i.e., practice, behavior of injecting drugs)

Sharing needles/syringes: refers specifically to the practice of sharing paraphernalia

Drug treatment entry: rates of individuals initiating treatment

Overdose: rates of overdose related to injecting drug behavior

Deaths: rates of death related to injecting drug behavior

## Discussion

This overview synthetized and critically appraised the methodological quality of 33 systematic reviews and meta-analyses published between 1998 and 2021 that assessed the effectiveness of harm minimization interventions (categorized into NSEP, OAT, behavioral/educational interventions, SCF or SIF, THN and combined approaches) in reducing risk behaviors associated with injecting drug use. These findings update and expand existing knowledge derived from previous overviews and may inform policy makers, practitioners and other stakeholders about the risk–benefit- ratio of these interventions for PWID, and underpin the development or improvement of guidance for implementation and upscale [57].

The inception of harm reduction policy emerged primarily as a response to the HIV/AIDS outbreak, shifting from an approach focusing on changing addictive behaviors to encompass broader public safety measures (e.g., reforming criminal policies) and harm reduction strategies to reduce the likelihood of individuals acquiring blood borne diseases, drug-related morbidity and fatality [58, 59]. These strategies should be implemented within a comprehensive public health framework, characterized by a pragmatic and humanistic approach and grounded on evidence-based evaluations [59, 60].

While over 90 countries had at least one harm reduction program implemented by [61], current debates revolve around the long-term cost-effectiveness of these interventions (or their combination) tailored to individual scenarios [59, 60]. As highlighted in our overview, despite numerous publications, the evidence seems primarily derived from low to moderate quality studies, with significant heterogeneity, inconsistent data (e.g., lack of standardized interventions, unclear adjustments for confounders) and relatively short follow-up periods. These factors hampered statistical evaluations in over 45% of the systematic reviews. Among the systematic reviews including statistical synthesis, the evidence was inconclusive in approximately 12% and partially in favor of the intervention (i.e., benefits found only for some outcomes) in around 40%.More than half of the systematic reviews presented low methodological quality, marked by differences in methods, outcomes, and transparency of report. Among the six high-quality systematic reviews, five reported at least one benefit in favor of the interventions, either NSEP [23], OAT [35, 52–54] or behavioral and psychosocial interventions [39]. The remaining two high-quality studies reported conflicting evidence [24, 42]. Previous studies have suggested that weak evidence, including studies with methodological flaws and high risk of bias, along with misleading and conflicting reports, can lead to biased recommendations and potentially distort decision-making. [62–64]. We advocate for the development of a core outcome set (COS), which entails a consensus-derived collection of outcomes and instruments to enable consistent measurement and reporting of harm minimization interventions [65, 66].

The World Health Organization (WHO) recommends targets for harm minimization interventions, including the distribution of 300 needles per PWID per year, the provision of OAT to more than 40 people per 100 PWID and viral hepatitis vaccination. However, these targets may fall short for meeting the needs of PWID daily or more frequently, estimated to correspond to 68.1% (95% CI 64.5–71.6%) on a global scale [67]. Moreover, 18% of PWID engage in receptive needle/syringe sharing at their last injection [68, 69]. A multinational study pointed to 33 (uncertainty interval [UI], 21–50) needle-syringes distributed via NSEP per PWID annually and 16 (UI, 10–24) OAT recipients per 100 PWID, significantly below recommendations [15, 68].

NSEP aims to advise PWID on safe injection practices, overdose prevention, and to facilitate referrals to treatment of drug use disorders. While evidence of NSEP on reducing HIV transmission and sharing needles/syringes can be graded as ‘sufficient’, it is often ‘inconsistent’ for HCV infection, other overall risk behaviors, and mortality [70–72]. This variability in findings may result from the intricacies of NSEP interventions, varied implementation and performance, differences across settings and geographical regions, all of which can lead to an over- or underestimation of the true effect of these programs in real-world [73–75]. These conclusions align with previous overviews by Fernandes et al. [11] (n = 13 systematic reviews) and Palmateer et al. [12] (n = 27 reviews), both reporting mixed results for NSEP. These authors highlighted that comprehensive harm reduction interventions at structural level and within multi-component programs may be associated with further significant benefits, likely due to the OAT component. In fact, according to the United Nations Office on Drugs and Crime and the WHO, interventions for reducing or eliminating HCV should be integrated, designed for simplified service delivery with a public health approach, and should include target HCV testing, care and treatments with direct-acting antivirals [76, 77]. The meta-analysis by Platt et al. 2017/2018 (high methodological quality) found a 74% reduction in the risk of HCV associated with the uptake of combined OAT with high coverage NSEP when compared to no OAT and low coverage or no NSEP (RR 0.26 [95% CI 0.07–0.89], based on studies presenting adjusted effect sizes). This effect size is larger than the one observed for OAT or NSEP alone (RR 0.50 [95% CI 0.40–0.63] and RR 0.79 [95% CI 0.39–1.61], respectively) [52–54]. OAT services aim to replace illicit drug use with medically prescribed, orally administered opiates such as buprenorphine or methadone. Their availability is increasing in prisons, as noted in our overview [78]. Nevertheless, additional research is needed to gain a better understanding of the impact of OAT in humanistic, social and economic outcomes.

Behavioral and psychosocial interventions are designed to address the psychological and social aspects of drug use. and are often delivered together with OAT by public institutions or non-governmental organizations, typically in outpatient settings [79]. Although with critically low or low methodological quality, the systematic reviews by Semaan et al., Copenhaver et al. and Meader et al. [37, 40, 43] concurred in suggesting that these interventions are effective in reducing risk behaviors. This effect was notable when associated with sexual exposure, suggesting that brief standard education can be a treatment option alongside other elective interventions in community outreach programs [22]. Our evidence gap map highlights the need for further evaluation of the impact of these interventions on more objective outcomes such as the transmission of blood-borne diseases, overdose rates, and drug-related fatalities. This is critical because behavioral outcomes are often self-reported, which may introduce reporting bias [12, 80].

In recent years, SCF or SIF have increasingly been implemented, particularly in areas characterized by frequent injecting in public places. They are designed to provide PWID with sterile injecting equipment, offer counselling services (before, during and after consumption), emergency care in the event of overdose, and facilitate referral to various forms of care [44, 45]. However, the evidence on SCF/SIF effectiveness is insufficient, primarily due to the lack of standardized outcomes and comparators. It may be necessary to consider intermediate outcomes (e.g., changes in practices) jointly with epidemiological data when evaluating interventions without a comparator. Other benefits such as number of diagnostics and immunization, referrals to detoxification, and decreased use of medical services, should be explored [48].

Our overview reveals a similar pattern of inconclusive findings from systematic reviews of low to moderate methodological quality on the impact of THN programs on PWID. THN programs’ primary aim is to reduce or prevent overdoses by providing users with training and naloxone kits. While previous studies demonstrated THN programs’ potential association with increased rates of overdose survival and successful overdose reversals [47, 81, 82], the review by Ansari et al. 2020 found mixed evidence [83].

Despite recent recommendations from WHO advocating decentralized, integrated and task-sharing services employing point-of-care viral load assays and reflex viral load testing to reduce HCV/HIV related harms in key populations, the evidence-base found in our study for point-of-care testing is inconclusive [77, 84].

Research should prioritize further methodologically robust primary studies on the impact of harm reduction interventions on HCV and specific subpopulations (e.g., prison). Studies focusing on standardization of outcomes related to drug overdose, mortality and injecting behaviors are essential to improve the evidence-base. Interrupted time series analyses have proven to be suitable to evaluate the effects of policy initiatives and could be used to assess the impact of harm minimization [85]. The literature on the long-term cost-effectiveness of these programs, particularly in community-based settings, remains heterogenous and somewhat inconclusive, which may be a barrier to program implementation and participant enrollment [83, 86]. Implementation of harm minimization may face external barriers (e.g., low political prioritization, inadequate coordination and integration, limited advocacy, and conflicting intersectoral policies). Additionally, stigma, ethical issues, and changes in drug consumption patterns pose challenges in participant engagement/acceptance, and program evaluation in real-world settings [87, 88]. This means that strategies and policies should be constantly adapted and innovated to address these evolving patterns and align with the culture and population characteristics [78, 89].

Our study has limitations. We did not assess the overlap of systematic reviews as it was not our primary objective. We used the AMSTAR-2, as it is a valid and reliable tool [20]. However, other approaches as the Risk Of Bias In Systematic Reviews (ROBIS) could yield similar results. The conclusions of the systematic reviews were considered as presented by authors, meaning that evidence may not be immediately transposed to different scenarios/settings and geographical regions. The critical appraisal of this overview may contain elements of subjectivity, which we tried to minimize by conducting a comprehensive systematic review (with no limitations on outcomes) according to international guidelines of conduct and report.

## Conclusions

The body of empirical findings synthetized in this overview, along with the evidence gap map, provides sufficient evidence to primarily support the role of OAT, NSEP, and especially their combination in reducing HIV/HCV transmission and some injecting risk behaviors among PWID. Further evaluations of objective outcomes, such as overdoses and drug-related fatality, should be explored in both short and long-term studies. Behavioral or psychological interventions were associated with reductions in sexual risk behaviors and, thus, should be considered as part of a structural-level approach. This approach focusses on strategies that aim to modify social conditions and arrangements by addressing the key drivers of HIV/HCV vulnerability through policy, legal, and environmental changes, as well as the empowerment of communities and groups for this population. Evidence on the effect of other harm minimization interventions, namely SCF or SIF and THN, as well as evidence in other settings or contexts remain insufficient. The impact of combined strategies is challenging to assess, since one or more components of interventions may contribute to the reduction of harmful outcomes. Therefore, further well-designed observational studies with standardized COS and consistent measurement of exposure to single interventions or the intensity of harm minimization interventions are needed to strengthen these findings.

## Disclosures

FAC works as a consultant for the World Health Organization, Regional Office for Europe. The author alone is responsible for the views expressed in this publication and these do not necessarily represent the decisions or the stated policy of the World Health Organization.

## Data Availability

All data is available upon reasonable request to the authors.
